# Impact of Donepezil Supplementation on Alzheimer’s Disease-like Pathology and Gut Microbiome in APP/PS1 Mice

**DOI:** 10.3390/microorganisms11092306

**Published:** 2023-09-13

**Authors:** Yuan Li, Mengyao Wu, Mengmeng Kong, Shaomei Sui, Qi Wang, Yan He, Jinsong Gu

**Affiliations:** 1 School of Biological Science and Technology, University of Jinan, Jinan 250022, China; 202121100281@stu.ujn.edu.cn (Y.L.); 17862859089@163.com (M.W.); bio_kongmm@ujn.edu.cn (M.K.); 2 Department of Neurology, The First Affiliated Hospital of Shandong First Medical University & Shandong Provincial Qianfoshan Hospital, Jinan 250014, China; 2620@sdhospital.com.cn (S.S.); 2715@sdhospital.com.cn (Q.W.)

**Keywords:** alzheimer’s disease, donepezil hydrochloride, gut microbiota, *Akkermansia*, molecular docking

## Abstract

Based on published information, the occurrence and development of Alzheimer’s disease (AD) are potentially related to gut microbiota changes. Donepezil hydrochloride (DH), which enhances cholinergic activity by blocking acetylcholinesterase (AChE), is one of the first-line drugs for AD treatment approved by the Food and Drug Administration (FDA) of the USA. However, the potential link between the effects of DH on the pathophysiological processes of AD and the gut microbiota remains unclear. In this study, pathological changes in the brain and colon, the activities of superoxide dismutase (SOD) and AChE, and changes in intestinal flora were observed. The results showed that Aβ deposition in the prefrontal cortex and hippocampus of AD mice was significantly decreased, while colonic inflammation was significantly alleviated by DH treatment. Concomitantly, SOD activity was significantly improved, while AChE was significantly reduced after DH administration. In addition, the gut microbiota community composition of AD mice was significantly altered after DH treatment. The relative abundance of *Akkermansia* in the AD group was 54.8% higher than that in the N group. The relative abundance of *Akkermansia* was increased by 18.3% and 53.8% in the AD_G group and the N_G group, respectively. Interestingly, *Akkermansia* showed a potential predictive value and might be a biomarker for AD. Molecular docking revealed the binding mode and major forces between DH and membrane proteins of *Akkermansia*. The overall results suggest a novel therapeutic mechanism for treating AD and highlight the critical role of gut microbiota in AD pathology.

## 1. Introduction

Alzheimer’s disease (AD) is a neurodegenerative disease with an insidious onset and a progressive course. With the progress of the disease, cognitive dysfunction and mental behavior abnormalities gradually appear, such as language, comprehension, orientation, reasoning, and judgment, eventually leading to loss of life force and workability. According to the World Alzheimer Report 2018, it is expected that by 2050, the number of AD patients will hit 152 million, which will lead to a profound burden on society [1]. Although AD has been studied for more than a century, its etiology and pathogenesis are not yet fully understood [2]. The main pathophysiological changes in the brain of AD patients are β-amyloid (Aβ) deposition to form “senile plaques” and abnormal phosphorylation of tau protein to form neurofibrillary tangles, which, in turn, lead to reduced synaptic strength, synapse loss, and neuronal degeneration [3]. In addition, oxidative stress, mitochondrial dysfunction, vascular factors, inflammatory damage, neurotransmitter deficiency, and comorbid pathologies are also found to be key factors in the AD process [4,5].

A main cholinergic hypothesis suggests that acetylcholine (Ach) deficiency in the prefrontal cortex and hippocampus is the most significant neurotransmitter change in AD patients, which is considered to be an important cause of cognitive decline in AD patients [6]. Its mechanism is associated with the degeneration and death of cholinergic neurons that are mainly located in the basal ganglia of Meynert, leading to a decrease in Ach in the brain, including the Papez circuit [7]. In addition, acetylcholinesterase (AChE) has been reported to play a role in the development of AD by promoting the assembly of amyloid fibrils related to Aβ deposition [8].

Donepezil hydrochloride (DH) is an AChE inhibitor with N-benzyl piperidine and isoindoline [9]. DH was approved by the Food and Drug Administration (FDA) as early as 1996 for the treatment of AD, with the inhibition of AChE activity, ensuing a rise in the Ach level and an improvement in AD as its suggestive mechanism [10]. Clinical and basic studies have confirmed that DH can reduce Aβ deposition in the AD brain, lower the levels of cytokines and inflammatory factors, increase the activities of catalase (CAT) and glutathione peroxidase (GSH-Px) to relieve oxidative stress response, repair the morphological structure of hippocampal neurons, and alleviate the loss of neurons [11,12].

Gut microbiota constitutes an important part of the microorganism in the human body, and there are complicated two-way communications between gut microbiota and multiple organs, among which the “microbiota-gut-brain axis” is associated with a variety of neurodegenerative diseases, involving neural, endocrine, and immune pathways [13]. Pathological damage in the brain of AD patients appears 15-20 years before clinical cognitive symptoms occur, and the lack of clear preclinical biomarkers is one of the reasons for the difficulty in the early diagnosis of AD. Therefore, finding biomarkers for preclinical diagnosis of AD and moving the time window of diagnosis and treatment forward have been hot spots in AD research [14,15]. The changes in gut microbiota are closely related to the occurrence and development of AD; even the alteration in gut microbiota may occur before AD pathology happens [16,17]. Several studies have shown that gut microbiota play an important role in the physiological processes of AD [18,19,20]. The composition of the gut microbiota in AD patients varies toward a distribution of bacteria associated with inflammation, and these changes may be related to the progression and severity of AD [21]. Compared with their young counterparts, old mice showed significantly increased abundance of inflammation-associated bacteria, such as *Escherichia-Shigella*, *Desulfovibrio*, and *Blautia* [22]. In addition, more significant blood–brain barrier (BBB) damage and reduced cerebral blood flow (CBF) were observed in the brains of older mice [23]. Our previous studies have also shown a significantly different community composition of gut microbiota between AD patients and their healthy caregivers [24]. Thus, for the exploration of the etiology and treatment of AD, it is of practical significance to analyze the characteristics of intestinal microbiota in AD by using multiple two-way communication systems, such as nervous, immune, endocrine, and metabolic systems [25].

In the present study, we investigated the intervention impact of DH on the pathology and gut microbiota of AD. The pathological changes in the brain and colon, sections of the prefrontal cortex, hippocampus, and colon were characterized. The gut microbiota after DH intervention was also analyzed, and molecular docking studies were performed to investigate the possible way for DH to interact with *Akkermansia*.

## 2. Materials and Methods

### 2.1. Animals

Female C57BL/6J mice (Beijing Vital River Laboratory Animal Technology Co., Ltd., Beijing, China) were stably crossbred with APP^swe^ × PS1^ΔE9^ double-transgenic male mice (Beijing Viewsoild Biotech Co., Ltd., Beijing, China). The offspring mice were used in subsequent experiments. All animals were kept under a standard environment of 23–26 °C and 50–70% humidity with a 12-h light/dark cycle and food and water supplied ad libitum [26]. Approximately 3 to 4 mm of mouse tail tip was nipped from the tip for extraction of DNA and ensuing genotyping using a PCR approach amplification according to Spark Mouse Tail Direct PCR Kit (SparkJade, Jinan, China) instructions. Genetically identified females were randomly divided into the AD group (*n* = 10) and AD gavage DH group (AD_G Group, *n* = 10), whereas the wild-type female littermates were randomly dichotomized into a normal group (N Group, *n* = 10) and a normal gavage DH group (N_G group, *n* = 10). From 4 months old, the N_G and AD_G groups were administered via gavage with DH (3 mg/kg/d) once a day for 24 consecutive weeks, while the N and AD groups received the same volume of PBS. All experimental procedures were approved by the Experimental Animal Ethics Committee of the First Affiliated Hospital of Shandong First Medical University (QFSYYPZ20200614).

### 2.2. Sample Collection

At the age of 10 months, fecal samples were collected from all animals and frozen at −80 °C for subsequent tests [27]. The mice were anesthetized with ether and treated with cardiac perfusion, and the intact brain and colon were removed after deprivation of food and water overnight. The brain tissue was cut into two halves. One half was stored at −80 °C for the analyses of enzyme activities, while the other half was fixed in 4% (*w*/*v*) paraformaldehyde for pathological analyses [28]. The colon was also fixed in 4% (*w*/*v*) paraformaldehyde for pathological analyses.

### 2.3. Aβ Immunohistochemical Test of Brain Sections and HE Staining of Colon Tissue

The hippocampus and prefrontal cortex were isolated from the paraformaldehyde-fixed brain, embedded in paraffin, and sectioned into micro slices. Rabbit Anti-beta Amyloid 1-42 antibody (No. bs-0107R) was used as the primary antibody, and Histostain TM-plus Kit (No. SP-0023) was used as the secondary antibody in the immunohistochemical procedure. The paraformaldehyde-fixed colon tissue was also embedded in paraffin, sectioned, and stained with hematoxylin and eosin. Aβ plaques in the prefrontal cortex and hippocampus were examined under a light microscope.

### 2.4. Determination of Superoxide Dismutase (SOD) and AChE Activities in the Brain and Colon

SOD activity detection kit (Solarbio, Beijing, China) was used to determine the activities of SOD in brain tissue, and an AChE activity detection kit (Solarbio, Beijing, China) was used to determine the AChE activities in the brain and colon tissues following the manufacturer’s instructions.

### 2.5. PCR Amplification and Product Purification

Total stool DNA was extracted according to the instructions of the TIANamp Stool DNA Kit (TIANGEN, Beijing, China). The forward primer was 341F (5′-CCTACGGGNGGCWGCAG-3′), and the reverse primer was 805R (5′-GACTACHVGGGTATCTAATCC-3′) [29]. The PCR program was set as follows: 98 °C, 30 s; 35 cycles of 98 °C, 10 s; 54 °C, 30 s; 72 °C, 45 s; and 72 °C, 5 min. The PCR product was purified using AMPure XT beads (Beckman Coulter Genomics, Danvers, MA, USA) and quantified by Qubit (Invitrogen, Carlsbad, CA, USA). Amplicon pools were used for sequencing, and the size and number of amplicon libraries were assessed on an Agilent 2100 Bioanalyzer (Agilent, Santa Clara, CA, USA) and an Illumina (Kapa Biosciences, Woburn, MA, USA) library quantification kit, respectively. The libraries were sorted on the NovaSeq PE250 platform.

### 2.6. 16S rRNA Sequencing Analysis

Samples were sequenced using an Illumina NovaSeq platform according to the manufacturer’s recommendations provided by LC-Bio. The paired-end sequences were assigned to the sample according to the unique barcode of the sample, and the barcode and primer sequences introduced into the library were removed. FLASH was utilized to merge the paired-end reads. Quality filtering on the raw reads was performed under specific filtering conditions to obtain high-quality clean tags according to Fqtrim (v.0.94). Chimeric sequences were filtered using Vsearch (v.2.3.4). DADA2 was used for demodulation to obtain the feature table and feature sequence. Diversity was calculated by normalizing to the same random sequence. Feature abundances were then normalized using the relative abundance of each sample according to the SILVA (release 132) classifier. All indicators of alpha diversity in our sample were calculated with QIIME2. Blast was used for sequence alignment, and the feature sequences were annotated with the SILVA database for each representative sequence.

### 2.7. Molecular Docking

The structure of DH was retrieved from the PubChem compound database (PubChem CID: 5741). The DH structure type was set to AD atom mode, and Auto Dock Tools (v.1.5.7) was used to set the number of roots and rotatable bonds of the ligand. The structure of mice AChE (PDB 2WLS, 2.6 Å) and *Akkermansia* outer membrane protein Amuc_1100 (PDB 6KNY, 2.1 Å) were obtained from the Protein Data Bank (PDB). The crystal structures of the two proteins were dewatered, hydrogenated, charged, and endowed with AD4 atom-type states, and the lattice energy calculation files were created using Auto Dock Tools. The active site box size was set as 60 × 70 × 60 Å, and the lattice spacing was 0.375 Å. The number of GA runs was set to 10, the population size to 150, the maximum number of evals to 2,500,000, and other parameters were set to default values. The search method was set to the Lamarck Genetic Algorithm (LGA). Auto Dock 4.0 was used for molecular docking. Auto Grid (v.1.5.7) and Auto Dock configuration (v.1.5.7) were run for auxiliary docking assistance. After completion of molecular docking, all structures produced by the same compound were clustered, and the all-atom Root Mean Square Deviation (RMSD) was set with a tolerance value of 2.0. Auto Dock 4.0 was used to check the docking energy of the molecule, and the structure with the lowest binding energy was chosen as the final result. PyMOL (v.2.5) was used to analyze the 3D structure complexes with the lowest energy [30]. Interactions between DH and protein receptors were mapped, including hydrogen bonds and hydrophobic interactions, which were observed using LigPlot+ (v.1.4) [31].

### 2.8. Statistical Analysis

When the data were not specified, all data were expressed as the mean standard deviation (mean ± SD). The Kruskal–Wallis rank sum test was used to compare the measurement data between multiple groups. The student’s *t* test or Welch *t* test (with unequal variance) was used for comparison between two groups. Image J (v.1.8.0) software was used for image quantitative analysis of Aβ plaques. *p* < 0.05 was considered statistically significant.

## 3. Results

### 3.1. Effects of DH on the Pathological Performance of the Brain and Colon in AD Mice

**Figure 1:**
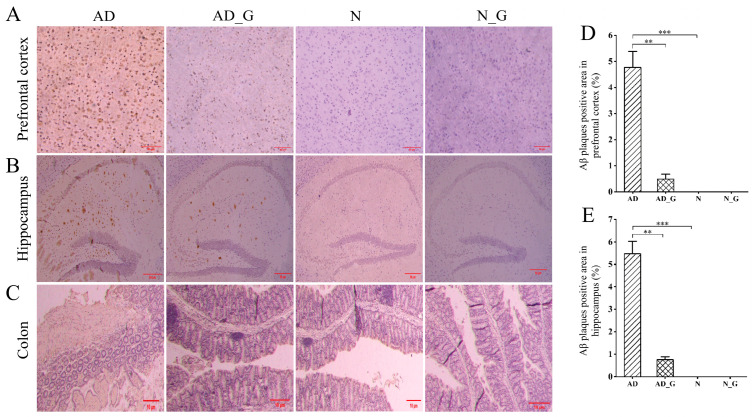
Effect of DH on pathological changes in AD mice. Aβ deposition in the (**A**) prefrontal cortex (Scale bar = 50 μm) and (**B**) hippocampus (Scale bar = 50 μm), and (**C**) colon pathomorphological changes (Scale bar = 10 μm). Proportion of Aβ plaques positive area in the (**D**) prefrontal cortex and (**E**) hippocampus. Values are expressed as the mean ± SD. Asterisks indicate significance levels as follows: **, *p* < 0.01; ***, *p* < 0.001.

The results for Aβ plaque deposition in the prefrontal cortex and hippocampus showed that there were evident Aβ plaques in the prefrontal cortex and hippocampus in the AD mice receiving 0.01 mL/g PBS (AD group) (Figure 1A,B), and the proportion of Aβ plaques in the whole area was significantly higher than that in the normal mice receiving the same amount of PBS (N group) (*p* < 0.001). Compared with the AD group, Aβ deposition in the prefrontal cortex and hippocampus in the AD mice that received 0.01 mL/g DH (AD_G group) was significantly decreased by 89.9% and 86.1%, respectively (Figure 1D,E). The structure of the colonic epithelium in the N group and normal mice that received 0.01 mL/g DH (N_G group) was normal, appearing uniformly arranged, complete, and continuous without inflammatory cell infiltration. In the AD group, part of the epithelial mucosa and intrinsic columnar structure disappeared, with fibrous response and infiltration of lymphocytes and plasma cells. In the AD_G group, the structure of the epithelium was intact, with no obvious fibrous or inflammatory response (Figure 1C).

### 3.2. SOD and AChE Activities in Brain and Colon

**Figure 2:**
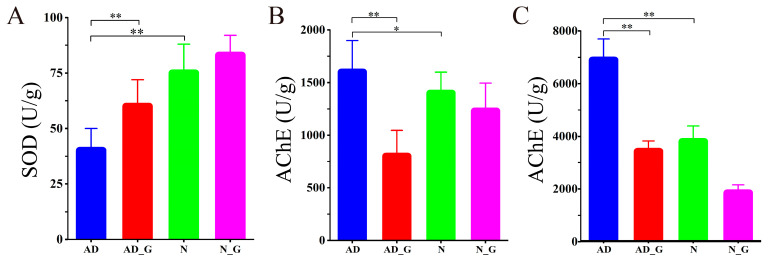
SOD and AChE activities in the brain and colon after DH administration. (**A**) Changes in SOD activity in brain. (**B**) Changes in AchE activity in brain and (**C**) colon. Data shown are means ± SD. For each index, the Student’s *t*-test was used to analyze the components for significant differences. Asterisks indicate significance levels as follows: *, *p* < 0.05; **, *p* < 0.01.

The SOD activity was significantly decreased by 42.5% in the AD group compared to the N group, while it was significantly increased by 32.1% in the AD_G group when compared with the AD group (Figure 2A). AChE activity in the brain was 10.9% higher in the AD group than in the N group, while it was 50.3% lower in the AD_G group than in the AD group (Figure 2B). In colon addition, AchE activity was significantly increased by 43.1% in the AD group compared with that in N group, while it was significantly decreased by 49.2% in the AD_G group than that in the AD group (Figure 2C).

### 3.3. Microbial Community Composition and Diversity Analysis of Gut Microbiota

**Figure 3:**
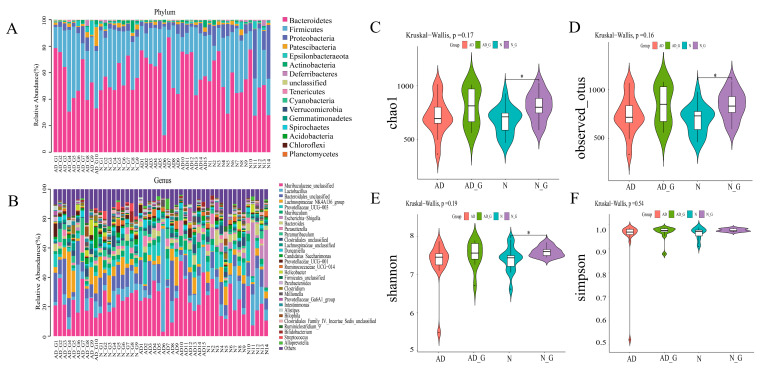
Impact of DH on the gut microbiota structure and diversity. Composition and distribution of the top species with the highest relative abundance at (**A**) phylum level and (**B**) genus level. Unclassified: No taxonomic information for this sequence was found in the database. (**C**) Chao1, (**D**) Observed_otus, (**E**) Shannon, and (**F**) Simpson indexes of alpha-diversity analysis. Data shown are means ± SD. For each index, Kruskal–Wallis was used to analyze the components for significant differences. Asterisks indicate significance levels as follows: *, *p* < 0.05.

To explore the impact of DH on gut microbiota, we sequenced the fecal samples of each group. The results of community composition showed that the microbial community changed significantly at the phylum level (Figure 3A). Compared with the N group, the relative abundance of Bacteroidetes and Actinobacteria was significantly increased, while the relative abundance of Firmicutes, Proteobacteria, Patescibacteria, Epsilonbacteraeota, and Deferribacteres was significantly reduced in the AD group.

The impact of DH on gut microbiota composition was further analyzed at the genus level. The results showed that compared with the N group, the relative abundance of *Lactobacillus*, *Prevotellaceae_UCG-003*, *Muribaculum*, *Parasutterella*, *Duncaniella*, *Ruminococcaceae_UCG-014*, *Millionella,* and *Alloprevotella* was significantly increased, and the relative abundance of *Lachnospiraceae_NK4A136_group*, *Candidatus_Saccharimonas*, *Helicobacter*, *Clostridium*, *Intestinimonas*, *Bilophila*, and *Ruminiclostridium_9* was significantly decreased in the AD group. However, DH treatment reversed the trend of changes at these genera in AD mice. In particular, compared with the AD group, the relative abundance of *Lactobacillus*, *Prevotellaceae_UCG-003*, *Muribaculum*, *Parasutterella*, *Duncaniella*, *Ruminococcaceae_UCG-014*, *Millionella*, and *Alloprevotella* was significantly decreased, and the relative abundance of *Lachnospiraceae_NK4A136_group*, *Candidatus_Saccharimonas*, *Helicobacter*, *Clostridium*, *Intestinimonas*, *Bilophila*, and *Ruminiclostridium_9* was significantly increased in the AD_G group.

The diversity analysis of intestinal microbiota showed that chao1 and observed_otus index were lower in the AD group than those in the N group and higher in the AD_G group than in the AD group. In addition, the chao1 and observed_otus indices of the N_G group were significantly higher than those of the N group (*p* < 0.05) (Figure 3C,D). Shannon index in the AD_G group was higher than that in the AD group, and in the N_G group, it was significantly higher than that in the N group (*p* < 0.05) (Figure 3E). However, the Simpson index did not differ significantly among the four treatments (Figure 3F).

### 3.4. Analysis of Differential Flora of Gut Microbiota

**Figure 4:**
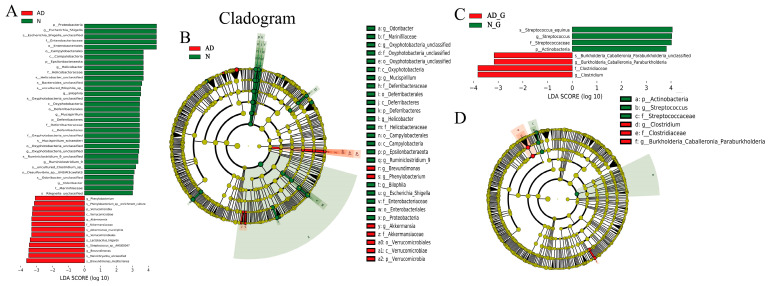
Effects of DH on differential flora. (**A**) LEfSe differential flora analysis between AD group and N group. (**B**) Cladogram of the LEfSe analysis of the AD group and N group. (**C**) LEfSe differential flora analysis between AD_G group and N_G group. (**D**) Cladogram of the LEfSe analysis of AD_G group and N_G group.

To further determine which bacteria were primarily responsible for the differences, LEfSe was used to perform a microbiota divergence analysis. A logarithmic linear discriminant analysis (LDA) score >3, *p* < 0.05 and profiling of LDA effect size analysis were used to identify important taxonomic differences between groups. In the N group, the dominant bacterial communities were Proteobacteria, *Escherichia coli*, *Shigella*, *Enterobacteriaceae*, *Epsilonbacteraeota*, *Helicobacter*, *Bilophila*, Deferribacterales, *Mucispirillum*, *Ruminococcaceae*, *Odoribacter*, and *Marinifilaceae*. Meanwhile, *Phenylobacterium*, *Phenylobacterium_sp_enrichment_culture*, Verrucomicrobia, *Akkermansiaceae*, *Lactobacillus Hilgardii*, *Streptococcus sp__AHSI00047*, and *Brevundimonas* were the dominant bacterial community in the AD group (Figure 4A,B). DH treatment altered the dominant bacterial community in both AD and N groups. Particularly, the dominant flora in the AD_G group was *Streptococcus* and *Actinomyces*, while the dominant flora in the N_G group was *Burkella* and *Clostridium* (Figure 4C,D).

**Figure 5:**
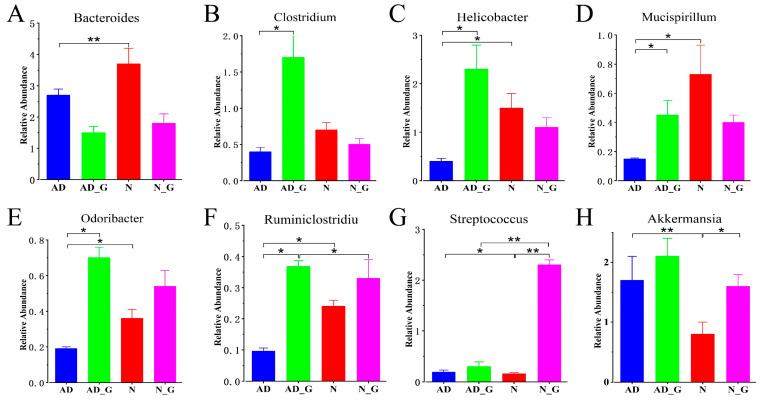
Relative abundance of differential bacterial genera among four groups. (**A**) *Bacteroides*, (**B**) *Helicobacter*, (**C**) *Mucispirillum*, (**D**) *Odoribacter*, (**E**) *Streptococcus*, (**F**) *Ruminiclostridiu*, (**G**) *Clostridium* and (**H**) *Akkermansia* in each group. Data shown were means ± SD. For each index, the Student’s *t*-test was used to analyze the components for significant differences. Asterisks indicated significance levels as follows: *, *p* < 0.05; **, *p* < 0.01.

The relative abundance analysis of eight bacterial genera in four groups showed that compared with the N group, the relative abundance of *Streptococcus* and *Akkermansia* was significantly increased by 16.0% and 54.8%, respectively (Figure 5G,H), while *Bacteroides*, *Helicobacter*, *Mucispirillum*, *Odoribacter,* and *Ruminiclostridium* were significantly decreased by 27.0%, 62.5%, 79.1%, 50%, and 60.9%, respectively, in the AD group (Figure 5A,C–F). In the AD_G group, the relative abundance of *Clostridium*, *Helicobacter* (Figure 5B), *Mucispirillum*, *Odoribacter,* and *Ruminiclostridium* was significantly increased by 75.5%, 80.0%, 64.3%, 72.1%, and 75.7%, respectively, compared with that in AD group. The relative abundance of *Akkermansia* was significantly increased by 53.8% in the N_G group compared with that in the N group.

### 3.5. Analysis of Disease Diagnostic Model and Phenotypic Prediction

**Figure 6:**
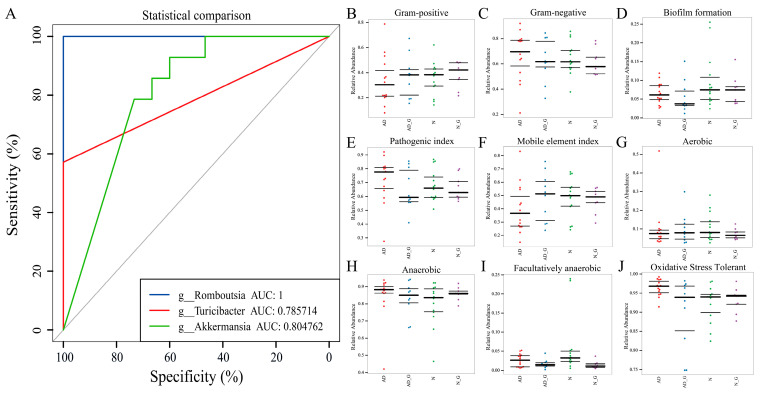
Disease diagnostic model and phenotypic prediction of intestinal flora. (**A**) Receiver operating characteristic curve (ROC) calculated using the predominant fecal microbial genera in discriminating AD from N group. Microbial communities were classified according to seven phenotypes by using bugbase: (**B**) Gram-positive, (**C**) Gram-negative, (**D**) biofilm formation, (**E**) pathogenic index, (**F**) mobile element index, (**G**) aerobic, (**H**) anaerobic, (**I**) facultative anaerobic, and (**J**) oxidative stress-tolerant. The three lines from the bottom up are maximum, median, and minimum values.

We evaluated the value of *Romboutsia*, *Turicibacter*, and *Akkermansia* as potential biomarkers. We used differential bacteria as a predictor to generate the area under the receiver operating characteristic curves to obtain the area under the curve (AUC), ranging from 0.5 to 1. The results showed that *Romboutsia* was the best discriminant predictor (AUC: 1), followed by *Akkermansia* as a moderate discriminant predictor (AUC: 0.8) (Figure 6A).

In addition, bugbase was used to classify the microbial communities according to seven types of phenotypes: Gram-positive, Gram-negative, biofilm-forming, pathogenic, mobile element-containing, oxygen-utilizing, including aerobic, anaerobic, facultatively anaerobic, and oxidative stress-tolerant. The results revealed that Gram-positive bacteria were at a low level in the AD group, while Gram-negative bacteria were at a dominant level in the AD group (Figure 6B,C), which is consistent with the result of the relative abundance of *Akkermansia* (Gram-negative bacteria) described above. The trend of biofilm formation in the N and N_G group was higher compared to the other two groups (Figure 6D), indicating that more bacteria were resistant to antibiotics and host immune defense mechanisms. The pathogenic index for AD had the highest trend among the four groups. Both the AD_G and N_C groups were significantly lower than that before gavage (Figure 6E), suggesting that DH supplementation is effective in reducing the harmful bacteria in the mice intestine. The mobile element indicator (Figure 6F) suggested that microbial evolution had the highest enhancement in the AD group, while DH treatment caused a decline in microbial evolution. The results of oxygen utilization (Figure 6G–I) showed that the average oxygen demand trend of the AD group was higher compared to other groups. Moreover, the highest trend of oxidative stress tolerance (Figure 6J) in the AD group indicated that AD increased the oxidative-damage-tolerant flora.

### 3.6. Interactions of DH with AchE and Amuc_1100

**Figure 7:**
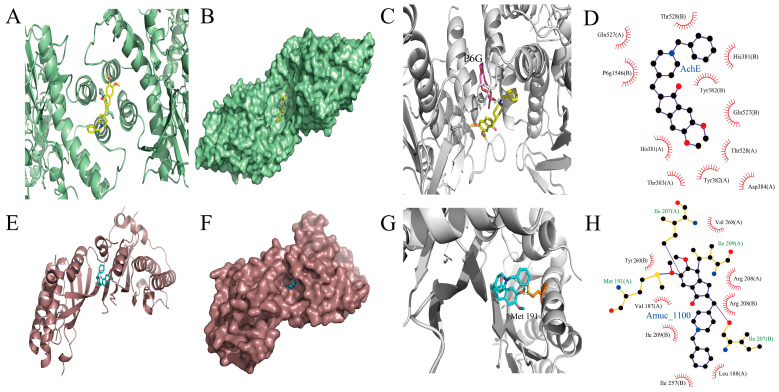
Molecular docking results of DH interactions with AchE and Amuc_1100. (**A**) Cartoon and (**B**) surface pattern of DH binding to AChE. (**C**) Interaction force and (**D**) two-dimensional display of the hydrophobic force between DH and AChE. (**E**) Cartoon and (**F**) surface pattern of DH binding to Amuc_1100. (**G**) Interaction force and (**H**) two-dimensional display of the hydrophobic force between DH and Amuc_1100.

The results of molecular docking of DH and AchE showed that DH was integrated into the groove of AChE (Figure 7A,B). A hydrogen bond was formed by the oxygen atom in coenzyme P6G-1546 in AChE with the ligand at a measured distance of 2.7 Å (Figure 7C). Furthermore, DH was encapsulated in the hydrophobic cavity of AChE and formed hydrophobic interactions with the surrounding amino acids, including Thr528 (A), Gln527 (A), His381 (B), P6g1546 (B), Tyr382 (B), Gln527 (B), His381 (A), Thr528 (A), Tyr382 (A), Thr383 (A), and Asp384 (A) (Figure 7D).

In addition, the docking results of DH and Amuc_1100, an outer membrane protein of *Akkermansia*, showed that DH could bind to the groove of Amuc_1100 (Figure 7E,F). The SD atom in the acceptor amino acid Met 191 in Amuc_1100 formed a hydrogen bond with DH, and the measured distance was 1.8 Å (Figure 7G). Furthermore, DH was encapsulated in the hydrophobic cavity of Amuc_1100 and formed hydrophobic interactions with surrounding amino acids, including Val268 (A), Tyr260 (B), Arg208 (A), Val187 (A), Arg208 (B), Ile209 (B), Leu188 (A), and Ile257 (B) (Figure 7H).

## 4. Discussion

Since the first case of AD was reported by the German psychiatrist Alois Alzheimer in 1906, many scholars have persisted in the research and practice in this field. No currently available treatment has been shown to reverse existing deficits or to arrest disease progression. Recently, many studies have shown a close link between gut microbes and AD disease [32,33,34]. Previous studies of molecular modeling on AD have shown that manipulating the gut microbiota can affect brain amyloid deposition [35]. As research progresses, the importance of gut microbes in AD becomes increasingly apparent. More evidence suggests that probiotics work through the microbial–gut–brain axis, which influences cognition in direct or indirect ways [36].

The extensive deposition of Aβ plaques in the prefrontal cortex and hippocampus is considered to be the most significant pathological hallmark of AD [37]. By observing Aβ deposition in the prefrontal cortex and hippocampus, we found that it was significantly increased in the AD group compared with the N group and significantly reduced in the AD_G group compared with the AD group. Previous studies have shown that the Aβ deposition found in AD mice and human brains was decreased after DH treatment [38], which is consistent with our data. Interestingly, we found that fibrosis and atypical inflammatory infiltration in colon tissue were reversed by DH treatment. A reduction in the AChE activity by DH induces a rise in the ACh level, which has been reported to improve colonic inflammation by inhibiting the release of inflammatory factors and increasing the expression of antimicrobial peptides [39,40].

Excessive reactive oxygen species (ROS) lead to oxidative stress (OS), and OS in the prefrontal cortex and hippocampus is considered to induce the production of Aβ [41]. SOD is one of the major enzyme components in detoxifying superoxide radicals. Its activity may indirectly reflect the severity of oxidative stress damage in AD [42]. Previous studies have reported the alleviation of Aβ deposition by increasing SOD activity and inhibiting AChE activity in AD mice and in vitro cells [43,44]. *Clostridium butyricum* has been demonstrated to increase SOD levels by regulating the p62-Keap1-Nrf2 signaling pathway and the intestinal microbial community [45]. *Odoribacter* is closely associated with ROS scavenging and increased SOD activity [46]. In addition, *Odoribacter* is a common short-chain fatty acid (SCFA)-producing member of the human gut microbiota [47]. Butyric acid has been demonstrated to increase SOD mRNA expression and eliminate excess ROS [48]. In addition, butyric acid also inhibits the release of inflammatory factors to alleviate inflammatory infiltration in the colon [49]. *Ruminiclostridium* is demonstrated to be butyric-acid-producing bacteria whose abundance is positively correlated with antioxidant activity and negatively correlated with inflammatory cytokines [50]. In this study, DH treatment significantly increased the activity of SOD in the brain and the abundance of *Clostridium*, *Odoribacter*, and *Ruminiclostridium*, indicating that modulating gut microbiota via DH enhanced brain antioxidant capacity.

Our research showed that the structure of the intestinal flora in AD mice was altered, with lower abundance and diversity than those in normal mice, while higher diversity was usually considered a sign of health [51].

Notably, we found that, as an indicator of aging [52], the ratio of Firmicutes/Bacteroidetes (F/B) decreased in the AD group, while the ratio was increased in the AD_G group. Firmicutes were significantly reduced in the AD mice at the phylum level, and the abundance of Firmicutes was increased in the AD_G group. The fermentation of dietary fiber by Firmicutes produces SCFAs, such as butyrate, propionic acid, and acetate. The disorder of intestinal flora leads to a decrease in the abundance of Firmicutes, which leads to a decrease in the production of SCFAs. However, SCFAs can reduce the production of pro-inflammatory factors, maintain the integrity of the BBB, and interfere with Aβ aggregation to a large extent to ameliorate AD [53,54]. Bacteroidetes were significantly increased in AD mice but reduced in the AD_G group. Bacteroidetes have the most complex polysaccharide capsule system among bacteria, consisting of at least eight different polysaccharides (PSA-PSH) that cause oxidative stress. AD is reported to cause intestinal inflammation and intestinal barrier damage [55], followed by Bacteroidetes and its metabolites entering blood circulation and being transported to the brain, resulting in damage to the central nervous system and aggravation of AD. It has been proved that the accumulation of lipopolysaccharide (LPS) promotes the formation of Aβ peptide fibers [56]. Based on these results, remission of the AD brain and colon pathology may be related to an increased abundance of Firmicutes and decreased abundance of Bacteroidetes, which lead to increased content of SCFAs in the intestine and reduced damage of LPS to the nervous system, indicating that the abundance of Firmicutes and Bacteroidetes plays an important role in the therapeutic effect of DH on AD.

In addition, many studies have also found that improvements in AD are accompanied by changes in dominant intestinal bacteria [57]. Our study showed that AD mice and normal mice had significantly different dominant gut microbiota, but DH treatment altered the dominant microbiota, suggesting that DH can reverse the group variability and dominance of gut microbiota to some extent.

*Bacteroides* are clinically important opportunistic pathogens present in most anaerobic infections [58]. A study showed enrichment in the abundance of *Bacteroides* in AD patients [59], which is consistent with our findings. *Helicobacter* is a genus of spiral-forming Gram-negative bacteria containing more than 35 species. DH reduced the abundance of *Helicobacter* in the N_G group and significantly increased it in the AD_G group, suggesting that DH caused a disruption in the *Helicobacter* in the diseased group while stabilizing the number and ability to affect this flora in the normal group.

A recent study reported that *Mucispirillum scheduler*, a low-abundance commensal bacteria carried by mice, can help the host resist *Salmonella typhimurium* infection by competing for nutrients, thus interfering with the establishment of *Salmonella* infection [60]. In this study, the abundance of *Mucispirillum* in the AD_G group was higher than in the AD group, which assisted the host in increasing its resistance to other complications.

Consistent with our findings, it has been reported that the abundance of *Odoribacter*, a member of SCFA-producing bacteria, was increased in the gut microbiota of AD mice with reduced Aβ deposition [61]. In addition, gut-microbiota-derived SCFAs, such as butyrate and propionic acid, can interfere with Aβ aggregation [62]. Therefore, *Odoribacter* may be involved in the clearance of Aβ deposits in the brain. A recent study showed that it is closely related to the microbiota–gut–brain axis [63]. *Streptococcus*, capable of clearing Aβ plaques by secreting neprilysin (NEP) [64], was more abundant in the N_G and AD_G groups than in the N and AD groups.

It has been reported that a significantly higher abundance of *Akkermansia* was found in AD mice compared with normal mice [65], which is consistent with our results. A recent study showed that *Akkermansia* can promote mucus production to increase the thickness of the intestinal mucus layer by promoting the differentiation of secretory mammalian intestinal epithelial cells (IECs) [66]. Meanwhile, *Akkermansia* promotes reductions in Aβ deposition in the prefrontal cortex of APP/PS1 mice [67]. In this study, DH increased *Akkermansia* abundance in both normal and AD mice. Based on the existing studies, we suggest that DH may promote the differentiation of IEC by increasing the abundance of *Akkermansia* in the intestine of AD mice, which promotes the synthesis and secretion of mucin, increases the thickness of the intestinal mucosa, restores the integrity of the intestinal barrier, alleviates the pathological changes in the colon, and reduces the deposition of Aβ.

Several studies have shown that *Akkermansia* plays a prebiotic role in chronic metabolic diseases, such as obesity and diabetes, but the relationship between *Akkermansia* and AD is not clear [68,69]. In the microbiota differential analysis, we found that the abundance of *Akkermansia* was significantly different between normal and AD mice and was increased by DH supplementation. Meanwhile, *Akkermansia* was demonstrated to be a potential biomarker for the diagnosis of AD via ROC curve analysis. Based on existing reports and our research, we suggested that *Akkermansia* may have the potential to be a biomarker for AD. The results of bacterial phenotype prediction showed significant differences between AD mice and normal mice, indicating that AD significantly altered the intestinal flora structure of mice, which corresponds to the results of community analysis described above. It has been reported that the majority of bacteria that make up the gut microbiome are anaerobic bacteria [70]. The increase in oxygen demand in the AD group may be the result of the disrupted structure of intestinal flora, reducing the abundance of anaerobic bacteria in the intestine. DH supplementation decreased the oxygen demand of the gut microbiota in AD mice, suggesting that DH regulates the disturbance of the gut microbiota.

Amuc_1100, an outer membrane protein of *Akkermansia*, is effective in improving chronic metabolic diseases, such as obesity and diabetes, promoting intestinal 5-HT synthesis and improving anxiety behavior [71]. We hypothesized that the underlying mechanism by which DH increased the abundance of intestinal *Akkermansia* may be that the interaction with Amuc_1100 promoted the proliferation of *Akkermansia* to reduce intestinal inflammation. The results of molecular docking showed that DH was in the hydrophobic cavity of protein macromolecules, and the interaction forces between DH and protein were mainly hydrogen bonding and hydrophobic interaction, which maintained the stability of Amuc_1100. In contrast to AChE, the interaction between DH and Amuc_1100 also had a hydrogen bond of less than 3 Å, multiple hydrophobic amino acid binding sites, and a common Tyr hydrophobic binding site, indicating that the docking results were effective and accurate and also suggesting that the two large molecular proteins were similar to each other to some extent.

It is reported that an alteration in gut microbiota is also likely to be related to other neurodegenerative diseases in addition to AD [72]. Several studies have reported that the abundance of *Bacteroides*, *Bifidobacterium*, and *Lactobacilli* decreases in old people [73,74]. Clinical research finds that the abundance of butyrate-producing microbes, including *Eubacterium rectale* and *Roseburia intestinalis*, is significantly decreased in Amyotrophic lateral sclerosis (ALS) patients [75]. Compared to research on gut microbiota in AD, more evidence exists on the relationship of gut microbes and their metabolite dysregulation in Parkinson’s disease (PD). It is reported that the abundance of *Bacteroidetes*, *Prevotella*, *Phascolarctobacterium*, *Eisenbergiella,* and *Gemella* is significantly different between PD and healthy individuals [76]. Further evidence suggests that Verrucomicrobiaceae (*Akkermansia muciniphila*) has increased abundance in PD patients [77]. Although AD is mainly manifested as cognitive impairment and PD is recognized as motor impairment, both AD and PD are the most common degenerative diseases of the central nervous system. AD and PD patients demonstrate a high degree of similarity in the changes in intestinal flora, which suggests that AD intervention therapy could learn from the successful experience of PD treatment.

To summarize, we here provided significant evidence that the abundance and diversity of gut microbiota were improved, and the dominant microbiota in AD and normal mice were altered through DH administration. Meanwhile, the abundance of beneficial bacteria was increased, while that of harmful bacteria was decreased. During this research, the potential of *Akkermansia* as a biomarker for the clinical diagnosis of AD was identified, and the similarity of DH interactions with AChE and Amuc_1100 was also demonstrated via molecular docking. And, a possible site of DH interaction with *Akkermansia* was provided. These findings provide valuable insight into the potential of DH to alleviate AD symptoms by affecting the gut microbiome, which provides new insight into the management of AD.

## Data Availability

Data are contained within the article.
